# Efficacy of canakinumab as first-line biologic agent in adult-onset Still’s disease

**DOI:** 10.1186/s13075-019-1843-9

**Published:** 2019-02-13

**Authors:** Giulio Cavalli, Alessandro Tomelleri, Giacomo De Luca, Corrado Campochiaro, Charles A. Dinarello, Elena Baldissera, Loreno Dagna

**Affiliations:** 1 Unit of Immunology, Rheumatology, Allergy and Rare Diseases, IRCCS San Raffaele Hospital, Vita-Salute San Raffaele University, Via Olgettina 60, 20132 Milan, Italy; 2 Vita-Salute San Raffaele University, Milan, Italy; 3 Department of Medicine, Radboud University Medical Center, Nijmegen, The Netherlands; 4 Department of Medicine, University of Colorado Denver, Aurora, CO 80045 USA

Adult-onset Still’s disease (AOSD) is a rare condition characterized by fever, arthritis, skin rash, and multi-organ inflammation. The pathogenesis is mediated by the pro-inflammatory cytokine interleukin (IL)-1β, as confirmed by the clinical efficacy of selective blockade. Anakinra, a recombinant inhibitor of the IL-1β receptor, currently represents the cornerstone of biologic therapy [1].

More recently, a monoclonal antibody blocking IL-1β, canakinumab, entered the clinical arena and became available for the treatment of AOSD. The efficacy of canakinumab in AOSD is being evaluated in a clinical trial (NCT022042939). At present, evidence from several case reports or series suggest good efficacy in AOSD (reviewed in [2, 3]): of note, in all published cases, canakinumab was used following failure of one or more biologics, including anakinra.

Here, we report the efficacy of canakinumab as a first-line biologic agent in AOSD. Four patients with severe DMARD-refractory AOSD received canakinumab (4 mg/kg/4 weeks) following failure of conventional treatment with corticosteroids and methotrexate. Patient characteristics and response to therapy are shown in Table 1. In all patients, treatment with canakinumab led to striking clinical responses, within days of initiation. Fever and skin rash disappeared first, followed by progressive improvement in arthritis. If present, inflammatory organ involvement also responded to treatment, as confirmed by resolution of pericardial inflammation and hepatosplenomegaly in two and one patients, respectively. Marked reductions in CRP, ESR, and serum ferritin mirrored the efficacy on clinical manifestations. Reduced disease severity allowed for robust tapering of corticosteroid therapy, which was discontinued in two patients and substantially reduced in two patients (Table 1).

**Table 1:** Patient characteristics and response to therapy

Clinical features	AOSD course	Therapy before CAN (mg)	Lab tests before CAN	Therapy after CAN (mg)	Lab tests after CAN	Response to CAN	Modified Pouchot score before CAN	Modified Pouchot score after CAN	Side effect
A, M, R, F, S	SD	PDN (15) MTX (20)	ESR 40 CRP 31.5 Ferritin 715	MTX (20)	ESR 12 CRP 4.2 Ferritin 140	Complete	7	1	None
A, M, R, F, HSM	SD	PDN (25) MTX (20)	ESR 45 CRP 8.2 Ferritin 880	PDN (5) MTX (15)	ESR 7 CRP 3.7 Ferritin 135	Complete	6	2	None
A, M, F, L, S	SD	PDN (10) MTX (10)	ESR 32 CRP 31.1 Ferritin 1324	–	ESR 12 CRP 5.7 Ferritin 98	Complete	5	1	None
A, M, F, P, R	SD	PDN (25) MTX (20)	ESR 57 CRP 17.4 Ferritin 1025	PDN (2.5) MTX (20)	ESR 9 CRP 2.1 Ferritin 119	Complete	6	1	None

AOSD duration indicates duration of disease before initiation of canakinumab (CAN). Disease manifestations: A arthritis; M myalgia; F fever; R rash; P pharyngitis; S serositis; L lymphadenopathies; HSM hepatosplenomegaly. Therapy before CAN indicates the treatment regimen that was being administered at the time of CAN initiation; therapy after CAN indicates the maintenance therapy that was being administered at the last follow-up visit. PDN prednisone; MTX methotrexate; SD systemic disease; ESR erythrocyte sedimentation rate (mm/1 h, normal values < 30 mm/1 h); CRP C-reactive protein (mg/L, < 6 mg/L); ferritin (ng/mL, 15–150 ng/mL). The modified Pouchot score for measuring AOSD disease activity evaluates clinical and laboratory manifestations and ranges from 0 to 12, with scores above 4 indicating active disease

Biologic therapy with IL-1 inhibitors should be instituted earlier in AOSD course for more favorable outcomes [2]. Both IL-1 blocking agents anakinra and canakinumab received EMA approval for the treatment of AOSD. Although anakinra and canakinumab block the same target, they have different mechanisms of action. Anakinra, a recombinant inhibitor of the IL-1 receptor, requires daily injections due to a short half-life of 6 h. Canakinumab, a fully human monoclonal antibody selectively blocking IL-1β, has a longer half-life and is administered monthly [4].

In this study, first-line biologic therapy of AOSD with canakinumab resulted in rapid and marked efficacy, ultimately leading to full clinical remissions in all patients and allowing for robust steroid-sparing effects. Canakinumab in AOSD is often used as a last line of treatment following failure of multiple other agents, including anakinra [2]. Early treatment is nevertheless advisable and may reduce chances of chronic disease and permanent damage [2, 5].

## Abbreviations

AOSD: Adult-onset Still’s disease
CRP: C-reactive protein
DMARD: Disease-modifying anti-rheumatic drug
ESR: Erythrocyte sedimentation rate
IL-1β: Interleukin-1β

## References

[1] Cavalli G, Franchini S, Aiello P, Guglielmi B, Berti A, Campochiaro C, Sabbadini MG, Baldissera E, Dagna L (2015). Efficacy and safety of biological agents in adult-onset Still's disease. Scand J Rheumatol.

[2] Junge G, Mason J, Feist E (2017). Adult onset Still’s disease-the evidence that anti-interleukin-1 treatment is effective and well-tolerated (a comprehensive literature review). Semin Arthritis Rheum.

[3] Colafrancesco S, Priori R, Valesini G, Argolini L, Baldissera E, Bartoloni E, Cammelli D, Canestrari G, Cantarini L, Cavallaro E (2017). Response to Interleukin-1 inhibitors in 140 Italian patients with adult-onset Still’s disease: a multicentre retrospective observational study. Front Pharmacol.

[4] Cavalli G, Dinarello CA (2015). Treating rheumatological diseases and co-morbidities with interleukin-1 blocking therapies. Rheumatology (Oxford).

[5] Pouchot J, Arlet JB (2012). Biological treatment in adult-onset Still’s disease. Best Pract Res Clin Rheumatol.

