# Correction to: Efficacy of canakinumab as first-line biologic agent in adult-onset Still’s disease

**DOI:** 10.1186/s13075-019-1850-x

**Published:** 2019-02-25

**Authors:** Giulio Cavalli, Alessandro Tomelleri, Giacomo De Luca, Corrado Campochiaro, Charles A. Dinarello, Elena Baldissera, Lorenzo Dagna

**Affiliations:** 1 Unit of Immunology, Rheumatology, Allergy and Rare Diseases, IRCCS San Raffaele Hospital, Vita-Salute San Raffaele University, Via Olgettina 60, 20132 Milan, Italy; 2 Vita-Salute San Raffaele University, Milan, Italy; 3 Department of Medicine, Radboud University Medical Center, Nijmegen, The Netherlands; 4 Department of Medicine, University of Colorado Denver, Aurora, CO 80045 USA

**Correction to: Arthritis Res Ther**

**https://doi.org/10.1186/s13075-019-1843-9**

Following publication of the original article [1], the authors reported an error in the spelling of Lorenzo Dagna’s name. The authors apologise for the error.

Incorrect spelling:

**Loreno Dagna**

Correct spelling:

**Lorenzo Dagna**

The original article [1] has been updated.
